# Exploring the relationships between wasting and stunting among a cohort of children under two years of age in Niger

**DOI:** 10.1186/s12889-021-11689-6

**Published:** 2021-09-21

**Authors:** Kristin Kohlmann, Christopher R. Sudfeld, Souna Garba, Ousmane Guindo, Rebecca F. Grais, Sheila Isanaka

**Affiliations:** 1 Department of Nutrition, Harvard T. H. Chan School of Public Health, Boston, USA; 2 Department of Global Health and Population, Harvard T. H. Chan School of Public Health, Boston, USA; 3 Epicentre, Niamey, Niger; 4 Epicentre, 14-34 Avenue Jean Jaurès, 75019 Paris, France

**Keywords:** Wasting, Stunting, Concurrence, Undernutrition, Growth, Child, Niger

## Abstract

**Background:**

Wasting and stunting, physical growth manifestations of child undernutrition, have historically been considered separately with distinct interventions at the program, policy, and financing levels despite similar risk factors, overlapping burdens and multiplicative risk of death when the conditions are concurrent. The aim of this study was to elucidate shared risk factors and the temporal relationship between wasting and stunting among children under 2 years of age in rural Niger.

**Methods:**

From August 2014 to December 2019, anthropometric data were collected every 4 weeks from 6 to 8 weeks to 24 months of age for 6567 children comprising 139,529 visits in Madarounfa, Niger. Children were defined as wasted if they had a weight-for-length Z-score < − 2 and stunted if they had a length-for-age Z-score < − 2 using the 2006 World Health Organization child growth standards. Parental, child, and socioeconomic risk factors for wasting and stunting at 6 and 24 months of age and the relationship between episodes of wasting, stunting and concurrent wasting-stunting were assessed using general estimating equations.

**Results:**

Half of children (50%) were female, and 8.3% were born low birthweight (< 2500 g). Overall, at 24 months of age, 14% of children were wasted, 80% were stunted and 12% were concurrently wasted-stunted. We found that maternal short stature, male sex, and low birthweight were risk factors for wasting and stunting at 6 and 24 months, whereas higher maternal body mass index and household wealth were protective factors. Wasting at 6 and 24 months was predicted by a prior episodes of wasting, stunting, and concurrent wasting-stunting. Stunting at 6 and 24 months was similarly predicted by prior episodes of stunting and concurrent wasting-stunting at any prior age but only by prior episodes of wasting after 6 months of age.

**Conclusions:**

These data support a complex and dynamic bi-directional relationship between wasting and stunting in young children in rural Niger and an important burden of concurrent wasting-stunting in this setting. Further research to better understand the inter-relationships and mechanisms between these two conditions is needed in order to develop and target interventions to promote child growth.

**Trial registration:**

ClinicalTrials.gov Identifier: NCT02145000.

## Background

The anthropometric measurement of children has been broadly used to identify two distinct manifestations of child under-nutrition. Wasting, defined by a low weight-for-length Z score (WLZ), is considered an indicator of acute undernutrition that is amenable to treatment and has long been the focus of humanitarian interventions that aim to reduce the immediate risk of death associated with wasting. Stunting, defined by a low length-for-age Z score (LAZ), is an indicator of chronic undernutrition and has traditionally been the focus of development organizations that seek to monitor linear growth faltering. These two forms of under-nutrition constitute a significant public health burden worldwide, with approximately 7% of children under-five wasted and 22% stunted in 2020 [1].

Wasting and stunting can co-exist in the same setting and occur within the same child [2]. Both manifestations of acute and chronic undernutrition share common risk factors, including infection, poor infant and young child feeding practices, inadequate diet and food insecurity, and poor maternal health and nutrition [3]. There is growing evidence of an inter-relationship between wasting and stunting, such that being wasted may increase the risk of subsequent stunting and vice versa [4, 5]. Importantly, evidence suggests that children with both wasting and stunting are at a greatly elevated risk of death [6]. Despite the potential for inter-relationships and common risk factors, academic research, programs, and policies have traditionally focused on treating wasting or preventing stunting in isolation [7, 8].

To better understand the relationship between wasting and stunting, we examined risk factors for wasting and stunting and assessed the temporal relationship between wasting and stunting over time using a longitudinal birth cohort in rural Niger.

## Methods

### Study setting

This study was conducted in the rural Madaraounfa Health District in the Maradi Region of south-central Niger. Niger has high levels of child mortality and is one the poorest countries in the world, ranking 189 out of 189 on the Human Development Index in 2018 [9]. The Maradi Region has the second highest under-five mortality in Niger with 166 deaths per 1000 live births. More than half (53.5%) of the children in the region are stunted and 19% are wasted at any given time [10]. The region is largely characteristic of the semi-arid Sahel. Household food production is linked to rain-fed agriculture, where staple crops such as millet and sorghum are harvested once per year. Each year, the decrease in food quantity and quality experienced in the months preceding the harvest and the concurrent increase in infectious illness, including diarrhea, pneumonia, and malaria, are associated with a seasonal increase in acute malnutrition among children under 5 years of age.

### Study design and population

We used prospective data from a double-blind, placebo-controlled, randomized controlled trial assessing the efficacy and safety of a bovine rotavirus pentavalent vaccine against severe rotavirus gastroenteritis (ClinicalTrials.gov Identifier: NCT02145000). Details of the parent study, including study enrollment, inclusion and exclusion criteria and study procedures have been described in detail elsewhere [11]. Briefly, in the parent study, a total of 6567 healthy infants were randomized to receive three doses of either vaccine or a placebo at 6, 10 and 14 weeks of age. Study children were followed on a weekly basis until 2 years of age to identify episodes of gastroenteritis, with scheduled growth monitoring conducted every 4 weeks. At enrollment into the trial, maternal anthropometry and household data were collected. Birthweight was attempted to be collected for all children within 72 h of birth. The parent trial included a nested, cluster randomized sub-study designed to test the effect of prenatal nutritional supplements on infant immune response [12]. Written informed consent was obtained from each mother and the child’s parent or legal guardian.

### Statistical analysis

Anthropometric indices were calculated according to the 2006 WHO growth standards [13]. Extreme values (<− 6 or > 6) were excluded. Wasting was defined as WLZ < -2, and stunting as LAZ < -2. As concurrent wasting-stunting has been increasingly recognized as an important issue and associated with an even higher increased risk of death than wasting or stunting alone [14], we further considered concurrent wasting-stunting (hereafter referred to as concurrence), defined as simultaneous WLZ < − 2 and LAZ < -2. Child, parental, and household characteristics of the study population were described using means and standard deviations for continuous variables and as percentages for categorical variables.

We described the prevalence of wasting, stunting, and concurrence using age-specific proportions by month from 6 to 8 weeks to 2 years of age, in the total population and by low birthweight status (< 2500 g) in the sub-sample of children for whom birthweight data was available. To identify predictors of wasting, stunting, and concurrence at 6 and 24 months of age, we considered child, parental, and household characteristics as potential risk factors using generalized estimating equations (GEE) with an independent correlation matrix and the log link to produce relative risk estimates and robust estimators of variance. To better understand the temporal relationship between episodes of wasting, stunting and concurrence, we used GEE models to examine the association of episodes of stunting, wasting, and concurrence at any prior age with the risk of being subsequently wasted or stunted at 6 and 24 months. In all analyses, we considered potential confounding by child sex, season of birth (lean season: June to October vs harvest season: November to May), season of visit (lean season: June to October vs harvest season: November to May), breastfeeding status, maternal literacy, paternal literacy, maternal short stature (< 155 cm), maternal body mass index (BMI), low maternal mid-upper arm circumference (MUAC,< 22 cm), maternal age, number of children under five in the household, whether the household was from a nomadic pastoralist tribe (Fulani or Tuareg vs Hausa or other), and household wealth (index constructed using principal components analysis of 10 items describing asset and livestock ownership, and housing quality). Final multivariate analyses were adjusted for any characteristic with a significant univariate association with the outcome at *P* < 0.2. Statistical analysis was carried out in Stata/IC version 15.1 (StataCorp LP, College Station, TX).

## Results

A total of 6567 children with 139,529 growth monitoring visits were included in the analysis. Fifty percent of the children in the study were female, 39.8% were born during the lean season (June to October), and 8.3% of infants were born low birthweight (< 2500 g) (Table 1). Mothers were on average 27 years old with a mean (SD) height of 157 (5.9) cm and a mean (SD) BMI of 22.0 (2.9) kg/m^2^ at the time of child enrolment into the trial. Less than half (46%) of households had access to improved sanitation, but the majority (91%) had access to an improved water source.

**Table 1:** Description of child, maternal and household characteristics of study participants

Characteristics	Percent (n) or Mean (SD) (***N*** = 6567)	
**Child Characteristics**		
Female	50.0% (3279)	
Birth during lean season (June–October)	39.8% (2614)	
Low birthweight (< 2500 g)	8.3% (460)	
Vaccine trial arm		
Vaccine	51.5% (3379)	
Placebo	48.5% (3188)	
**Maternal Characteristics**		
Mother literate	32.9% (2161)	
Maternal height, cm	157.3 (± 5.9)	
Maternal age, years	26.7 (±6.6)	
Maternal MUAC, cm	26.1 (±2.6)	
Maternal BMI, kg/m^2^	22.0 (± 2.9)	
**Household Characteristics**		
Improved sanitation in household^a^	46.6% (3060)	
Improved water in household^b^	91.1% (5980)	
Number of children under 5y in household		
One child	12.8% (841)	
Two children	42.0% (2755)	
Three children	22.5% (1480)	
≥ Four children	22.7% (1489)	

^a^Improved sanitation was defined as using flush toilets, improved pit latrines, or simple latrines with slabs

^b^Improved water sources included piped running water, cased wells, and protected dug wells

**Fig. 1:**
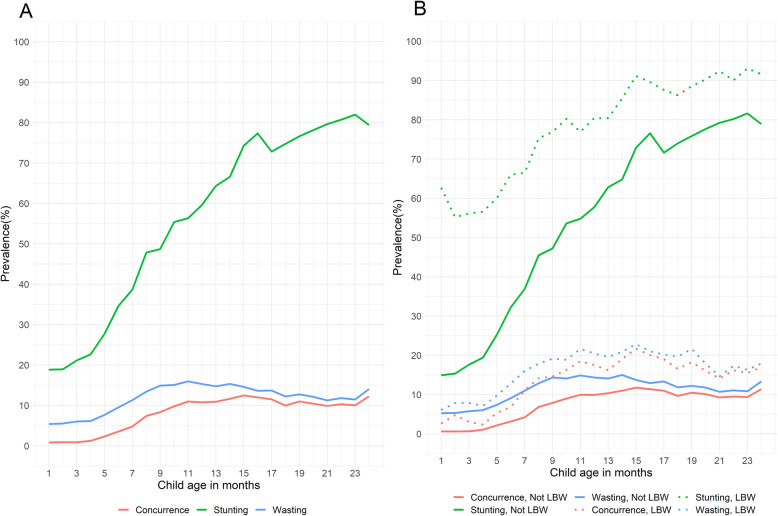
Wasting, stunting and concurrent wasting-stunting prevalence from 6 to 8 weeks up to 24 months of age. Panel A depicts growth outcomes of the entire study population. Panel B depicts growth outcomes by low birthweight status for the sub-sample of children with birthweight data. WHO growth standards (2006) were used to calculate Weight-for-Length Z-score (WLZ) and Length-for-Age Z-score (LAZ) for ages 1 to 24 months. Wasting was defined as WLZ < -2 SD, stunting as LAZ < -2 SD, and concurrence as simultaneous WLZ < -2 and LAZ < -2

We evaluated the prevalence of wasting, stunting, and concurrence of wasting and stunting from 6 to 8 weeks to 2 years of age (Fig. 1, Panel A). The prevalence of wasting was lowest for the first 6 months (5–10%), before increasing to 14–16% from 9 to 18 months, decreasing to 11–12% from 19 to 23 months of age, and then increasing again to 14% at 24 months. Stunting prevalence more consistently increased over time from 17% at 6 weeks to 80% at 24 months of age. The prevalence of concurrence of wasting and stunting increased over time, peaking at 15 months of age (12.5%), following the trends in wasting prevalence. The prevalence of stunting was consistently higher among low birthweight infants compared to non-low birthweight infants throughout follow-up, whereas the prevalence of wasting and concurrence of wasting and stunting both emerged higher among low birthweight infants as compared to non-low birthweight infants after approximately 10 months of age (Fig. 1, Panel B).

We also examined parental, child, and socioeconomic risk factors for wasting and stunting at 6 and 24 months of age (Table 2). Maternal short stature was a common risk factor and household wealth a common protective factor for child wasting at both 6 and 24 months of age. Further, higher maternal age was associated with increased risk of wasting at 6 months of age, while being born in the lean season decreased the risk for wasting at 6 months of age. In contrast, being born in the lean season increased the risk of wasting at 24 months of age. Other factors that were associated with increased risk of wasting at 24 months of age included being interviewed in the lean season and being breastfed. In addition, higher maternal BMI decreased the risk of wasting at 24 months.

**Table 2:** The association between child, parental, and household characteristics with the risk of wasting and stunting at 6 months and 24 months

	Wasting at 6 months (***n =*** 4990)		Wasting at 24 months (***n =*** 5334)		Stunting at 6 months (***n*** = 4987)		Stunting at 24 months (***n*** = 5333)	
	RR (95%CI)	aRR (95%CI)	RR (95%CI)	aRR (95%CI)	RR (95%CI)	aRR (95%CI)	RR (95%CI)	aRR (95%CI)
**Child characteristics**								
Female	0.95 (0.81–1.13)		0.70 (0.61–0.80) **	0.70 (0.61–0.80) **	0.80 (0.74–0.86) **	0.79 (0.74–0.86) **	0.91 (0.88–0.93) **	0.90 (0.88–0.93) **
Lean season birth	0.58 (0.48–0.70)**	0.63 (0.49–0.82) **	1.37 (1.20–1.56) **	1.19 (1.02–1.38) *	1.03 (0.95–1.11)		1.00 (0.97–1.02)	
Lean season visit	1.52 (1.29–1.81)**	1.11 (0.88–1.40)	1.39 (1.22–1.59) **	1.25 (1.08–1.45) **	0.96 (0.89–1.04)		0.99 (0.96–1.01)	
Currently breastfed	0.72 (0.29–1.80)		1.35 (1.09–1.66) **	1.33 (1.08–1.64) **	1.30 (0.72–2.36)		1.11 (1.07–1.15) **	1.11 (1.07–1.15) **
**Parental Factors**								
Mother literate	0.90 (0.75–1.09)		0.81 (0.70–0.94)**	0.89 (0.76–1.05) +	0.96 (0.89–1.05)		0.96 (0.93–0.99) **	0.97 (0.94–1.01) +
Father literate	0.91 (0.76–1.07)		0.88 (0.77–1.01) +	0.99 (0.85–1.15)	0.91 (0.84–0.98) *	0.95 (0.88–1.02) +	0.97 (0.95–1.00) *	1.00 (0.97–1.03)
Mother short stature (< 155 cm)	1.15 (0.96–1.37) +	1.18 (0.99–1.41) +	1.18 (1.03–1.36) *	1.23 (1.07–1.41) **	1.48 (1.38–1.60) **	1.49 (1.38–1.61) **	1.19 (1.16–1.22) **	1.20 (1.17–1.23) **
Maternal BMI at enrollment (per unit increase in kg/m^2^)	0.99 (0.95–1.02)		0.92 (0.89–0.94) **	0.93 (0.90–0.95) **	0.97 (0.96–0.99) **	0.97 (0.96–0.99) **	0.99 (0.98–0.99) **	0.99 (0.98–1.00) **
Maternal MUAC < 22 cm	1.28 (0.80–2.04)		1.33 (0.93–1.91) +	0.98 (0.68–1.40)	1.11 (0.89–1.38)		1.08 (1.01–1.16) *	1.00 (0.93–1.08)
Maternal Age	1.02 (1.01–1.03) **	1.02 (1.01–1.03) **	1.01 (1.00–1.02)		1.00 (0.99–1.00)		1.00 (1.00–1.00) *	1.00 (1.00–1.00)
**Household Factors**								
Number of children under 5 years in household	1.02 (0.96–1.07)		1.03 (0.98–1.08)		1.02 (1.00–1.05) +	1.04 (1.01–1.06) **	1.00 (0.99–1.01)	
Nomadic pastoralist tribe	1.20 (0.81–1.80)		1.45 (1.07–1.95) *	1.24 (0.92–1.66) +	1.17 (0.99–1.40) +	1.09 (0.92–1.30)	1.06 (0.99–1.13) +	1.03 (0.96–1.10)
Household wealth index								
Poorest	1.00	1.00	1.00	1.00	1.00	1.00	1.00	1.00
Poor	1.01 (0.79–1.30)	0.99 (0.77–1.26)	1.04 (0.86–1.25)	1.05 (0.87–1.26)	1.01 (0.91–1.13)	0.98 (0.88–1.10)	0.98 (0.94–1.02)	0.97 (0.94–1.01) +
Middle	0.85 (0.65–1.10)	0.83 (0.64–1.08) +	0.96 (0.78–1.17)	1.01 (0.83–1.23)	0.95 (0.85–1.07)	0.95 (0.85–1.06)	0.98 (0.94–1.02)	0.98 (0.94–1.02)
Wealthy	0.75 (0.57–0.99)*	0.75 (0.57–0.99) *	0.78 (0.63–0.96) *	0.81 (0.65–1.01) +	0.84 (0.74–0.95) **	0.85 (0.75–0.96) **	0.95 (0.92–1.00) *	0.95 (0.91–0.99) *
Wealthiest	0.78 (0.60–1.02) +	0.81 (0.62–1.05) +	0.74 (0.59–0.91) **	0.82 (0.66–1.02) +	0.80 (0.71–0.91) **	0.83 (0.74–0.94) **	0.91 (0.87–0.95) **	0.92 (0.88–0.96) **

** *p* < 0.01, * *p* < 0.05, + *p* < 0.20

Risk factors for stunting at 6 and 24 months of age included male sex and maternal short stature, whereas protective factors included higher maternal BMI and higher household wealth. Breastfeeding also increased the risk of stunting at 24 months of age.

In the sub-sample where birthweight data was available, we found that low birthweight (< 2500 g) was associated with increased risk of wasting at 6 and 24 months of age (RR = 1.48 (95%CI: 1.11–1.97) and RR = 1.36 (95%CI: 1.07–1.72),respectively), stunting at 6 and 24 months of ae (RR = 2.05 (95%CI: 1.87–2.24) and RR = 1.16 (95%CI: 1.12–1.20), respectively), and concurrence at 6 and 24 months (RR = 2.16 (95%CI: 1.40–3.32) and RR = 1.49 (95%CI: 1.17–1.91), respectively).

We finally explored the temporal relationship of wasting and stunting in the cohort (Table 3). We found that wasting at 6 and 24 months was consistently predicted by wasting, stunting, and concurrence at from 2 to 21 months of age. The only exceptions were that wasting at 2 months of age and concurrence at 2 and 4 months of age were not significantly associated with wasting at 24 months of age. The risk of stunting at 6 and 24 months was consistently predicted by stunting and concurrence from 2 to 21 months of age, but only prior episodes of wasting after 6 months of age predicted stunting at 24 months of age.

**Table 3:** Relationship of prior wasting, stunting, and concurrent wasting-stunting with the risk of wasting and stunting at 6 and 24 months of age

	Risk of wasting (Multivariate analysis)					Risk of stunting (Multivariate analysis)			
	6 months (***N =*** 4990)			24 months (***N =*** 5334)		6 months (***N =*** 4987)		24 months (***N =*** 5333)	
	aRR (95%CI)		P	aRR (95%CI)	P	aRR (95%CI)	P	aRR (95%CI)	P
**Wasted status by age in months**									
2 m	2.89 (2.31–3.61)		< 0.001	1.04 (0.78–1.40)	0.79	1.20 (1.02–1.40)	0.02	0.98 (0.91–1.04)	0.46
4 m	5.36 (4.50–6.39)		< 0.001	1.34 (1.04–1.72)	0.02	1.16 (0.99–1.35)	0.06	1.03 (0.98–1.09)	0.23
6 m				1.96 (1.62–2.37)	< 0.001			1.09 (1.05–1.14)	< 0.001
9 m				2.25 (1.93–2.64)	< 0.001			1.05 (1.01–1.09)	0.02
12 m				3.04 (2.62–3.54)	< 0.001			1.09 (1.05–1.12)	< 0.001
15 m				3.64 (3.16–4.20)	< 0.001			1.12 (1.08–1.15)	< 0.001
18 m				3.91 (3.40–4.50)	< 0.001			1.09 (1.05–1.13)	< 0.001
21 m				4.24 (3.70–4.85)	< 0.001			1.09 (1.05–1.13)	< 0.001
**Stunted status by age in months**									
2 m	1.36 (1.10–1.67)		< 0.01	1.38 (1.18–1.62)	< 0.001	2.61 (2.34–2.81)	< 0.001	1.20 (1.17–1.23)	< 0.001
4 m	1.32 (1.07–1.61)		< 0.01	1.40 (1.20–1.64)	< 0.001	3.61 (3.35–3.89)	< 0.001	1.27 (1.24–1.30)	< 0.001
6 m				1.59 (1.37–1.85)	< 0.001			1.31 (1.28–1.34)	< 0.001
9 m				1.58 (1.34–1.85)	< 0.001			1.47 (1.42–1.52)	< 0.001
12 m				1.87 (1.55–2.26)	< 0.001			1.70 (1.62–1.77)	< 0.001
15 m				2.22 (1.75–2.80)	< 0.001			2.33 (2.16–2.51)	< 0.001
18 m				2.16 (1.71–2.73)	< 0.001			2.65 (2.45–2.87)	< 0.001
21 m				1.99 (1.55–2.53)	< 0.001			4.02 (3.57–4.53)	< 0.001
**Concurrent wasting-stunting status by age in months**									
2 m	3.73 (2.59–5.37)		< 0.001	0.94 (0.45–1.97)	0.87	2.18 (1.83–2.60)	< 0.001	1.20 (1.15–1.25)	< 0.001
4 m	5.42 (4.10–7.16)		< 0.001	0.90 (0.44–1.84)	0.77	2.43 (2.09–2.81)	< 0.001	1.17 (1.10–1.25)	< 0.001
6 m				1.91 (1.45–2.51)	< 0.001			1.19 (1.15–1.24)	< 0.001
9 m				2.59 (2.18–3.08)	< 0.001			1.20 (1.17–1.23)	< 0.001
12 m				3.17 (2.71–3.70)	< 0.001			1.21 (1.18–1.24)	< 0.001
15 m				3.84 (3.34–4.43)	< 0.001			1.18 (1.15–1.21)	< 0.001
18 m				3.96 (3.45–4.54)	< 0.001			1.18 (1.14–1.21)	< 0.001
21 m				4.09 (3.56–4.69)	< 0.001			1.18 (1.15–1.22)	< 0.001

The adjusted model for wasting at 6 months controlled for season of birth, season of visit, maternal short stature, maternal age, and household wealth. The adjusted model for wasting at 24 months controlled for child sex, season of birth, season of visit, breastfeeding status, maternal literacy, paternal literacy, maternal short stature, maternal BMI, maternal low MUAC, whether the household was from a nomadic pastoralist tribe, and household wealth. The adjusted model for stunting at 6 months controlled for child sex, paternal literacy, maternal short stature, maternal BMI, number of children in the household, whether household was from a nomadic pastoralist tribe, and household wealth. The adjusted model for stunting at 24 months controlled for child sex, breastfeeding status, maternal literacy, paternal literacy, maternal short stature, maternal BMI, maternal low MUAC, maternal age, whether household was from a nomadic pastoralist tribe, and household wealth

## Discussion

This analysis used longitudinal data from a large cohort of children in rural Niger to explore the relationship between child wasting, stunting, and concurrent wasting-stunting during the first 2 years of life. The prevalence of stunting was 80% by 24 months of age and the prevalence of wasting was highest at 16% at 11 months of age. We determined that maternal short stature, male sex, and low birth weight were shared risk factors for wasting and stunting at 6 and 24 months of age, whereas higher maternal BMI and higher household wealth were common protective factors. We also found that prior stunting and concurrence of stunting and wasting increased the risk of subsequent wasting and stunting. Prior episodes of wasting at any age also predicted subsequent wasting, but only prior episodes of wasting after 6 months of age were associated with increased risk of subsequent stunting.

The age-specific patterns of the prevalence of wasting and stunting in our cohort were aligned with data from other low- and middle-income countries. Schoenbuchner et al. found an increase in wasting prevalence peaking around 1 year before decreasing slightly in the Gambia [5, 15], similar to our observation that wasting prevalence peaked and remained high from 9 to 18 months of age. We also saw a steady increase in stunting prevalence over the first 2 years of age, which was also described by longitudinal cohorts in the Gambia, India and Guatemala, a pooled analysis of 7 African countries, and global data from cross-sectional demographic health surveys [3, 5, 15, 16]. By 2 years of age, 80% of the children in our study were stunted, which is higher than national estimates for Niger [17]. However, the high rate of concurrent wasting and stunting were similar to that reported using nationally representative survey data from Niger (8, 95%CI 7.2–8.9%) [2]. We found the prevalence of concurrent wasting and stunting gradually increased until 15 months of age before decreasing again, following the trend in wasting prevalence. We do note, however, that in addition to age patterns, greater understanding of the onset or incidence of these conditions should also inform the timing of effective preventive interventions. Recent pooled analysis of longitudinal cohorts in low- and middle-income settings showed that, in contrast to age-specific prevalence data, both wasting and stunting incidence were in fact highest between birth and age 3 months, new evidence that underscores the need for early intervention [16, 18].

We examined risk factors for wasting and stunting at 6 and 24 months and found that low birth weight was associated with increased risk of wasting, stunting, and concurrence during the first 2 years of life. This finding is consistent with prior studies from Africa and Asia [19–23]. Previous reports estimate as much as 30% of childhood wasting and 20% of stunting originate in the fetal period [24], while a large pooled analysis of the causes of child growth failure similarly concluded that maternal, prenatal, and at-birth characteristics were the strongest predictors of growth failure among children in low- and middle-income countries [25]. The association between low birthweight and future growth outcomes in this analysis underscores the importance of pre-pregnancy and pregnancy interventions to reduce the risk of child wasting and stunting.

Our findings also highlight the importance of maternal nutritional status (stature) in predicting the risk of both wasting and stunting, which is in agreement with the results of several other studies [19, 20, 23, 25–28]. In our study, maternal short stature increased the risk of stunting at both 6 and 24 months, which is potentially mediated through an increased risk of prematurity and fetal growth restriction [29, 30]. Nevertheless, a recent analysis of 35 longitudinal cohorts in 15 low- and middle-income countries determined that birth outcomes only partially mediated the relationship between maternal stature and BMI and child linear growth and therefore other mechanisms are possible [25]. The authors therefore suggested that maternal nutritional status may also affect postnatal growth through breastmilk quality, or reflect family poverty, genetics, or lifestyle/diet. Similar to other studies, we also found that being male and being currently breastfed were associated with an increased risk of wasting and stunting [19, 23, 31, 32]. Unlike some of the previous literature, we did not find that maternal education was associated with the risk of wasting or stunting at 6 or 24 months of age [19, 23]. This, however, could be due to the limited variability in this factor in our population: 77% of mothers were illiterate.

We also found that prior wasting was a risk factor for subsequent stunting with the magnitude of the risk of stunting generally similar with episodes of wasting after 6 months of age. Further, the magnitude of the risk of stunting with prior episodes of wasting was generally much smaller with greater time between assessments. This finding is generally consistent with a large pooled analysis of 35 longitudinal cohorts in 15 low- and middle-income countries that also reported a consistent, exposure-response relationship between higher mean WLZ and faster linear growth velocity in the following 3 months [25]. Richard et al. similarly reported that wasting at 6–11 months or 12–18 months was associated with a decreased LAZ at 18–24 months of age, while more temporally distant wasting that occurred at 0–5 months was not associated with length deficits during any period up to 24 months [4]. In the Gambia, Schoenbuchner et al. considered episodes of wasting which occurred 3 months prior and found that wasted children had 3.2 (95%CI: 2.7–3.9) higher odds of being stunted when they were 20–24 months old than non-wasted children [5]. Previous studies among malnourished children also suggest that recovery of linear growth may be delayed until adequate weight is recovered which provide further support that proximal episodes of wasting may contribute to impaired linear growth [33, 34].

Our results also indicate that previous stunting was associated with increased risk of wasting. Our findings support earlier research that stunted children aged 17–21 months had 1.5 (95%CI: 1.3–1.7) higher odds of being wasted 3 months later [5]. The mechanisms underlying the relationship of stunting and wasting are unclear, however, the association of stunting with subsequent wasting may be mediated through negative influences on body composition and long-term physiological changes [35]. Overall, the bi-directional associations between stunting and wasting observed in our study indicate a complex inter-relationship between the two forms of undernutrition and additional research is needed to elucidate the mechanisms that link stunting and wasting.

Our study design and data analysis have several strengths and limitations. These longitudinal data are the first to be reported from Niger and allowed us to obtain repeated monthly anthropometric measures on a large sample of children which provided benefits over studies that examine age-wise patterns in cross-sectional surveys. We also collected data on a comprehensive set of predictors including parental, child and socioeconomic indicators. While this allowed for a comprehensive set of variables to be included in multivariate analysis, residual confounding from unmeasured covariates cannot be ruled out. One limitation of our study is that we used only anthropometry to characterize under-nutrition. Body composition, including lean and fat tissue was not assessed in the parent trial, which could have provided further insight into the physiological and functional inter-relationships of wasting and stunting. Additionally, there is evidence that gestational age can influence postnatal growth, however, accurate estimates of gestational age were not available in this cohort.

## Conclusion

Our results support a dynamic inter-relationship between childhood wasting and stunting in the context of rural Niger. Importantly, we provide evidence for the bi-directional association between the two conditions, where wasting is associated with near-term stunting and stunting is predictive of wasting. The historical emphasis placed by policymakers and public health programs on either wasting or stunting reductions has likely missed important opportunities and synergies to improve child nutrition and growth more broadly. Our findings should motivate research and programs that evaluate integrated approaches to address wasting and stunting simultaneously in order to reduce the substantial burden of child undernutrition in low-and middle-income countries and support achievement of global child mortality and sustainable development goals.

## Data Availability

The dataset analyzed during the current study are available from the corresponding author on reasonable request.
